# Laboratory Diagnosis of COVID-19: Current Issues and Challenges

**DOI:** 10.1128/JCM.00512-20

**Published:** 2020-05-26

**Authors:** Yi-Wei Tang, Jonathan E. Schmitz, David H. Persing, Charles W. Stratton

**Affiliations:** a Cepheid, Danaher Diagnostic Platform, Shanghai, China; b Department of Pathology, Microbiology and Immunology, Vanderbilt University Medical Center, Nashville, Tennessee, USA; c Cepheid, Sunnyvale, California, USA; Boston Children's Hospital

**Keywords:** COVID-19, SARS-CoV-2, specimen type, molecular testing, serology, result interpretation

## Abstract

The COVID-19 outbreak has had a major impact on clinical microbiology laboratories in the past several months. This commentary covers current issues and challenges for the laboratory diagnosis of infections caused by severe acute respiratory syndrome coronavirus 2 (SARS-CoV-2). In the preanalytical stage, collecting the proper respiratory tract specimen at the right time from the right anatomic site is essential for a prompt and accurate molecular diagnosis of COVID-19. Appropriate measures are required to keep laboratory staff safe while producing reliable test results. In the analytic stage, real-time reverse transcription-PCR (RT-PCR) assays remain the molecular test of choice for the etiologic diagnosis of SARS-CoV-2 infection while antibody-based techniques are being introduced as supplemental tools. In the postanalytical stage, testing results should be carefully interpreted using both molecular and serological findings. Finally, random-access, integrated devices available at the point of care with scalable capacities will facilitate the rapid and accurate diagnosis and monitoring of SARS-CoV-2 infections and greatly assist in the control of this outbreak.

## Abstract (precis)

The COVID-19 outbreak has had a major impact on clinical microbiology laboratories in the past several months. This commentary covers current issues and challenges for the laboratory diagnosis of infections caused by severe acute respiratory syndrome coronavirus 2 (SARS-CoV-2). In the preanalytical stage, collecting the proper respiratory tract specimen at the right time from the right anatomic site is essential for a prompt and accurate molecular diagnosis of COVID-19. Appropriate measures are required to keep laboratory staff safe while producing reliable test results.

## TEXT

The identification by U.S. public health officials of presumptive COVID-19 cases believed to be due to community transmission of this infection brings into sharp focus the importance of the laboratory diagnosis of infection caused by severe acute respiratory syndrome coronavirus 2 (SARS-CoV-2) (1–5). The current recommendations for laboratory diagnosis of COVID-19 from the CDC are that clinicians coordinate this testing with local public health authorities and/or the CDC. The preferred testing method is the real-time reverse transcription-PCR (RT-PCR) test (6–8) similar to that developed for the diagnosis of SARS-CoV (9, 10). Viral cultures are not recommended. This commentary addresses current issues for the laboratory diagnosis of COVID-19 that must be understood by clinicians, clinical microbiology laboratories, and public health authorities.

## 

### Preanalytical issues.

**(i) Initial respiratory tract specimen collection for diagnosis and screening of patients with COVID-19 pneumonia.** Within 5 to 6 days of the onset of symptoms, patients with COVID-19 have demonstrated high viral loads in their upper and lower respiratory tracts (11–14). A nasopharyngeal (NP) swab and/or an oropharyngeal (OP) swab are often recommended for screening or diagnosis of early infection (9, 12, 15). A single NP swab has become the preferred swab as it is tolerated better by the patient and is safer to the operator. NP swabs have an inherent quality control in that they usually reach the correct area to be tested in the nasal cavity. Wang et al. have just reported that OP swabs (*n* = 398) were used much more frequently than nasal swabs (*n* = 8) in China during the COVID-19 outbreak; however, the SARS-CoV-2 RNA was detected in only 32% of OP swabs, which was significantly lower than the level in nasal swabs (63%) (16). While collection/testing of both nasal and OP swabs, either as independent specimens or together within a single aliquot of viral transport medium, might be an attractive option under normal circumstances, institutions must also consider the potential stress that this pandemic places on national/international supply chains. In this light, another excellent reason to limit testing with NP swabs is to prolong supplies of flocked swabs and/or transport media. However, as we understand more about respiratory and oral contact routes of transmission, we may learn that patients with pharyngitis as a dominant initial presenting symptom can be adequately sampled via the OP route.

In order to properly obtain an NP swab specimen, the swab must be inserted deeply into the nasal cavity. Patients will likely flinch, but that means the swab has hit the target. Swabs should be kept in place for 10 s while being twirled three times. Swabs should have flocked nontoxic synthetic fibers, such as polyester, as well as synthetic nylon handles (17). Collecting an NP/OP swab specimen may carry a theoretical risk of transmitting SARS-CoV-2, particularly if airborne transmission is demonstrated as the investigation of the COVID-19 outbreak continues (18). If personal protective equipment (PPE) cannot be utilized due to scarcity of such PPE, other means of collecting upper respiratory tract specimens will be needed (18). One alternative option for collecting an upper respiratory tract specimen to evaluate patients with suspected COVID-19 pneumonia is a self-collected saliva specimen (19–22). Should the supply of swabs become scarce, other nonflocked swabs and transport media have been cleared equivalently by the Food and Drug Administration (FDA) under an emergency use authorization (EUA), but head-to-head comparisons are lacking currently.

After collection, swabs should be placed in viral (universal) transport medium for rapid transportation to the clinical microbiology laboratory, ideally under refrigerated conditions (17). It should be noted, however, that in some cases, saliva/NPs/OPs may miss early infection and that in later infection, the main site of replication may have shifted to the low respiratory tract. Repeated testing or obtaining lower respiratory tract specimens may be required. Moreover, other respiratory viral pathogens such as influenza and respiratory syncytial viruses must be ruled out. In many ways, COVID-19 highlights the key difference between analytic and clinical sensitivities, that is, the ability of an assay to detect a pathogen when it is present in a clinical specimen versus the ability of a test to identify a patient’s overall infected status. The latter, of course, reflects various other factors that include the specimen site and method of collection, in conjunction with the burden of organism as a function of anatomic location, disease severity, and time symptomatic (and variability of these factors from individual to individual). Repeated testing may be particularly important if a patient has a clinical picture of viral pneumonia, a potential exposure history, and/or radiographic findings (chest computed tomography [CT] or magnetic resonance imaging [MRI] scan) consistent with COVID-19 pneumonia. Equally challenging are how the results of a single undetected result should impact decisions regarding patient quarantine and social distancing, in particular when the patients themselves are health care providers (including clinical laboratory staff). Serology, as discussed in the postanalytical section, may assist in such situations.

**(ii) Late detection and monitoring of patients with severe COVID-19 pneumonia.** Ideally, sputum sampling or bronchoalveolar lavage should be used for collecting lower respiratory tract specimens as they have yielded the highest viral loads for the diagnosis of COVID-19 (18, 23). A recent study revealed that samples bronchoalveolar lavage (BAL) fluid yielded the highest SARS-CoV-2 RNA rate although this study did not compare/evaluate results from NP swabs (16). Patients who present with severe pneumonia and acute respiratory distress syndrome may require emergent intubation as well as respiratory isolation in a negative-pressure room. If possible, a lower respiratory tract sputum specimen should be collected during the intubation procedure. Alternatively, sputum and/or bronchoalveolar lavage fluid specimens can be collected after intubation (9, 11).

However, some patients with COVID-19 pneumonia have demonstrated high viral RNA loads of SARS-CoV-2 in fecal material (24, 25) as well as delayed shedding from the respiratory tract (4, 18) late in their clinical course. Enteric involvement previously has been seen in patients with severe novel coronavirus infections (9, 26–32). In four such studies, SARS coronavirus was isolated from stool cultures (26, 28, 31). In another study, SARS coronavirus was demonstrated inside enterocytes by electron microscopy (30). Thus, aside from direct respiratory sampling, the preferred method for detecting SARS-CoV-2 in advanced COVID-19 cases may be a rectal swab and real-time RT-PCR (9, 26–28, 30–32).

**(iii) Safety measures for specimen processing for PCR processing and testing.** Processing of respiratory specimens should be done in a class II biological safety cabinet (6, 9, 10), although some laboratories would argue that biosafety level three (BSL-3) work procedures should be used and that the safety cabinet should be in a negative-pressure room within the laboratory such as that used for mycobacterial cultures. For nucleic acid extraction before real-time RT-PCR is performed, the specimen should be transferred to lysis buffer under this BSL-2 cabinet. The lysis buffer should contain a guanidinium-based inactivating agent as well as a nondenaturing detergent. Indeed, the buffers included in common commercial extraction platforms, such as the bioMérieux easyMAG or Qiagen EZ1, do contain guanidium/detergents and are able to inactivate any viable coronavirus (33–35). Similarly, universal transport medium that includes guanidinium salt is available from Merlin Biomedical (Xiamen, China) (http://www.chinamerlin.com/en/index.php?p=products_show&id=166&s_id=&c_id=68&lanmu=2). Because this test is a reverse transcription method, the saliva/swabs used to collect the clinical specimens should be quickly added to lysis buffer to disinfect the specimen as well as to stop degradation of the coronavirus RNA (6, 9, 10). The clinical specimens/swabs should not be heated to 56°C for 30 min as evidence suggests that this process may also degrade the coronavirus RNA even as it inactivates viable coronavirus (9, 36).

Moreover, self-enclosed systems integrating nucleic acid extraction, amplification, and detection such as ID NOW (Abbott, San Diego, CA) (37, 38), cobas Liat (Roche Molecular Systems, Pleasanton, CA), and GeneXpert (Cepheid, Sunnyvale, CA) (39), when available and meeting local regulatory requirements for SARS-CoV-2 testing, will be very useful. Once the clinical specimen in viral transport medium is transferred into a cartridge in a class II biosafety cabinet, the cartridge is sealed. Many of these random-access sealed devices are suitable for point-of-care testing for local hospitals and clinics without biosafety cabinets. In this situation, the specimen collector in appropriate protective gear (splash guard/goggles, mask, gloves, and disposable laboratory coat) could directly transfer the specimen into detection cartridges at bedside or in a location without a class II biosafety cabinet, and the closed cartridge could be safely placed on an instrument for testing. However, spills of transport solution during transfer to these cartridge-based tests should be avoided, and if they occur, decontamination should be performed as appropriate.

### Analytical issues.

**(i) Assay selection.** Immunoassays have been developed for rapid detection of SARS-CoV-2 antigens or antibodies. These rapid point-of-care immunoassays are generally lateral flow assays, but high-throughput immunoanalyzer versions are also in development for population-level screening. Such lateral flow assays have been developed for detecting antigens such as the SARS-CoV-2 virus or for detecting antibodies (IgM and IgG) against COVID-19.

Rapid antigen lateral flow assays would theoretically provide the advantage of a fast time to result and low-cost detection of SARS-CoV-2 but are likely to suffer from poor sensitivity early in infection, based on the experience with this method for influenza (Flu) viruses (40–44). Monoclonal antibodies specifically against SARS-CoV-2 have been under development, and several rapid antigen assays are being developed (45). There is concern that, given the variability of viral loads in COVID-19 patients, antigen detection may miss cases due to low infectious burden or sampling variability.

Serology measures the host response to infection and is an indirect measure of infection that is best utilized retrospectively. Serological methods are rapidly being developed and have proven to be useful in confirming past COVID-19 (25). Serology previously has had an important role in the epidemiology of SARS (46) and other coronavirus outbreaks (47). Rapid lateral flow assays for both IgM and IgG antibodies undoubtably will play an important role in the COVID-19 outbreak and should allow the burden of infection, the role of asymptomatic infections, the basic reproduction number, and the overall mortality to be determined. However, IgM responses are notoriously nonspecific, and given the weeks required to develop specific IgG responses, serology detection is not likely to play a role in active case management except to diagnose/confirm late COVID-19 cases or to determine the immunity of health care workers as the outbreak progresses. Cell culture is not recommended for diagnostic purposes.

**(ii) Assay selection for molecular detection of SARS-CoV-2.** Random-amplification deep-sequencing methods played a major role in the initial identification of SARS-CoV-2 (48–52). Deep sequencing molecular methods such as next-generation sequencing and metagenomic next-generation sequencing will continue to be needed to determine future mutations of SARS-CoV-2 but are currently impractical for diagnosing COVID-19. Most of the molecular diagnostics being developed for the diagnosis of COVID-19 involve real-time RT-PCR assays, including those from the U.S. Centers for Disease Control and Prevention (53), Charité Institute of Virology in Berlin, Germany (7, 54), and Hong Kong University (21, 55). Other molecular methods are being developed and evaluated worldwide and include loop-mediated isothermal amplification, multiplex isothermal amplification followed by microarray detection, and CRISPR (clustered regularly interspaced short palindromic repeats)-based assays (56).

**(iii) Target selection for real-time RT-PCR assays.** A real-time RT-PCR method is recommended for molecular testing (6, 8–10). A major advantage of real-time RT-PCR assays is that amplification and analysis are done simultaneously in a closed system to minimize false-positive results associated with amplification product contamination. There are a number of coronaviruses that cause respiratory and intestinal infections in humans (8, 57). Among these coronaviruses are a group of SARS-like bat coronaviruses, including both SARS-CoV and SARS-CoV-2, that comprise a unique clade under the subgenus *Sarbecovirus* (57, 58). Coronaviruses have a number of molecular targets within their positive-sense, single-stranded RNA genome that can be used for PCR assays (6, 7, 57, 58). These include genes encoding structural proteins, including envelope glycoproteins spike (S), envelope (E), transmembrane (M), helicase (Hel), and nucleocapsid (N) (57–59). In addition to the genes that encode structural proteins, there are species-specific accessory genes that are required for viral replication. These include RNA-dependent RNA polymerase (RdRp), hemagglutinin-esterase (HE), and open reading frame 1a (ORF1a) and ORF1b (7, 53–55, 57, 58). In the United States, the CDC recommends two nucleocapsid protein targets (N1 and N2) (53) while WHO recommends first-line screening with an E gene assay followed by a confirmatory assay using the RdRp gene (7). Chan et al. have just developed and compared the performance of three novel real-time RT-PCR assays targeting the RdRp/Hel, S, and N genes of SARS-CoV-2. Among them, the COVID-19-RdRp/Hel assay had the lowest limit of detection *in vitro* and higher sensitivity and specificity (59). However, it is likely that well-optimized targets will arise from a number of viral genomic locations since assay performance is usually dictated by the reagent design, not the target itself, since the viral genes are present in equal copy numbers.

To avoid potential cross-reaction with other endemic coronaviruses as well as potential genetic drift of SARS-CoV-2, at least two molecular targets should be included in the assay. Various investigators in different countries have used a number of these molecular targets for real-time RT-PCR assays. In the United States, the CDC has selected two loci in the nucleocapsid gene as the two-target assay appears to be performing well (53). One study utilized two sequence regions (open reading frame 1b and a nucleocapsid protein) that are highly conserved among sarbecoviruses for initial real-time RT-PCR testing (6). Another study in Hong Kong, China, used two targets for its RT-PCR assay; the first used the nucleocapsid for screening followed by confirmation by the open reading frame 1b (55). In Germany, two molecular targets (envelope and RNA-dependent RNA polymerase) have been selected (7). In China, at the time of manuscript preparation, several molecular devices had received urgent approval (8). To date, there has been no indication that any one of the sequence regions used offers a unique advantage for clinical diagnostic testing. However, the ideal design would include at least one conserved region and one specific region to mitigate against the effects of genetic drift, especially as the virus evolves within new populations.

In the United States, regulatory issues have complicated the development and implementation of laboratory-developed molecular tests for the diagnosis of COVID-19. On 29 February 2020, the FDA issued new guidance for laboratories to be able to develop and implement COVID-19 molecular diagnostic tests prior to obtaining EUA. Laboratories are required to submit an EAU to the FDA within 15 business days after validation. Moreover, the validation must include the specimen types (e.g., nasopharyngeal, oropharyngeal, or saliva) that are to be used clinically. Although these new regulatory burdens did not prohibit the development of molecular laboratory testing for the diagnosis of COVID-19, they did create a lot of extra work. At the time of writing, the U.S. FDA had granted quite a few EUAs (https://www.fda.gov/medical-devices/emergency-situations-medical-devices/emergency-use-authorizations#coronavirus2019; accessed 28 March 2020).

### Postanalytical issues.

**(i) Interpretation of molecular results.** In the United States, initially if both of two targets in the CDC assay (nucleocapsid proteins N1 and N2) test positive, a case is considered to be laboratory confirmed (53). A cycle threshold (*C_T_*) value of less than 40 is defined as a positive test, while a *C_T_* value of 40 or more is defined as a negative test. A *C_T_* value of <40 for only one of the two nucleocapsid protein (N1 and N2) is defined as indeterminant and requires confirmation by retesting (53). Currently, in China for the assays with three targets, positives for two or more targets are considered positive (60). Although some correlations have been revealed, viral loads determined by real-time RT-PCR assays should not be used yet to indicate COVID-19 severity or to monitor therapeutic response (11–13, 61, 62). However, low *C_T_* values indicating high viral loads may be used as an indication of transmissibility (18, 63).

**(ii) Test of cure and test of infectivity.** Monitoring patients with resolution of COVID-19 pneumonia may also be important in terms of when they should be released from isolation and discharged. If discharged patients are still shedding viable coronavirus, they are likely to infect other people (27). Therefore, self-quarantine for up to 1 month has been recommended in some cases. NP and OP swabs may not be sufficient for either test of cure or test of infectivity (64), but this needs further investigation. One approach to test of cure has been to demonstrate two consecutive negative real-time RT-PCR tests from rectal swabs; this suggestion is based on the fact that SARS-CoV-1 was cultured from stool during the 2002-2003 SARS outbreak (26, 28, 31), and SARS-CoV-2 has been cultured from stool during the COVID-19 outbreak (16). Thus, a rectal swab that is positive by real-time PCR testing suggests that this patient may be shedding viable SARS-CoV-2 in their stools, thereby remaining infectious (16, 24–28, 30–32). However, a very recent study on 20 serial COVID-19 patients indicated that infectious virus was not isolated from stool samples in spite of high virus RNA concentrations (14). The correlation of RT-PCR positivity in stool with recovery of live virus from the same samples remains to be fully investigated.

**(iii) Serology of COVID-19.** Members of the coronavirus family have four structural proteins: the spike (S), membrane (M]), envelope (E), and nucleocapsid (N) proteins. Two of these proteins appear to be important antigenic sites for the development of serological assays to detect COVID-19. Serological methods have focused on detecting serum antibodies against S proteins from the coronavirus spike (47). The coronavirus envelope spike is responsible for receptor binding and fusion and determines host tropism and transmission capability (57, 58). S proteins are determined by the S gene and are functionally divided into two subunits (S1 and S2). The S1 domain is responsible for receptor binding while the S2 domain is responsible for fusion. SARS-CoV and SARS-CoV-2 bind to human angiotensin-converting enzyme 2, which is found on human respiratory cells, renal cells, and gastrointestinal cells (57, 65, 66). The other protein that appears to be an important antigenic site for the development of serological assays to detect COVID-19 is the N protein, which is a structural component of the helical nucleocapsid. The N protein plays an important role in viral pathogenesis, replication, and RNA packaging. Antibodies to the N protein are frequently detected in COVID-19 patients (67, 68), suggesting that the N protein may be one of the immunodominant antigens in the early diagnosis of COVID-19 (69).

As mentioned above, rapid lateral flow assays for antibodies (IgM and IgG) produced during COVID-19 have been developed (70). Seroconversion occurred after 7 days of symptomatic infection in 50% of patients (14 days in all) but was not followed by a rapid decline in viral load (14). Serological methods, when available, will play an important role in the epidemiology of COVID-19 and in determining the immune status of asymptomatic patients but are unlikely to play any role in screening or for the diagnosis of early infections (14, 67, 68). However, serology may be useful for confirming the diagnosis of COVID-19 (25).

### Concluding remarks.

The ongoing, unprecedented outbreak of COVID-19 globally has emphasized the importance of the laboratory diagnosis of human coronavirus infections in order to limit the spread as well as to appropriately treat those patients who have a serious infection. This commentary has addressed current issues regarding such testing for SARS-CoV-2. For example, an NP rather than OP swab is recommended for early diagnosis or screening because it provides higher diagnostic yields, is better tolerated by the patient, and is safer for the operator. An NP swab can be combined with an OP swab to increase sensitivity but requires twice the number of swabs. Should the NP swabs become scarce, self-collected saliva or nasal washes could be used as an alternative specimen type for epidemiological screening and for the “worried well,” who are asymptomatic persons with no exposure history who wish to be tested just to be sure they are not infected. NP swabs would then be reserved for hospitalized patients; those who test negative may need deep sputum or BAL fluid samples collected. The importance of repeated testing or the use of bronchoscopy in patients with severe illness should the first screening test be negative must be understood. The role of rectal swabs in testing patients with late infection or as a test of infectivity/cure is currently not well studied but needs urgent attention. Equally unappreciated is the need for broad screening/testing with molecular testing and/or serological testing in order to determine the true mortality rate as well as other epidemiological markers. Finally, the importance of rapid development of integrated, random-access, point-of-care molecular devices for the accurate diagnosis of SARS-CoV-2 infections cannot be overemphasized. These short-turnaround-time (STAT) tests will be very important for real-time patient management and infection control decisions, especially when other less infectious forms of pneumonia are present and respiratory isolate resources are scarce. These assays are safe, simple, and fast and can be used in local clinics and hospitals that already have the needed instruments and that are responsible for identifying and treating such patients.

## References

[1] Del Rio C, Malani PN 28 2 2020 COVID-19—new insights on a rapidly changing epidemic. JAMA doi:10.1001/jama.2020.3072. 32108857

[2] Fauci AS, Lane HC, Redfield RR 2020 Covid-19—navigating the uncharted. N Engl J Med 382:1268–1269. doi:10.1056/NEJMe2002387. 32109011 PMC7121221

[3] Guan WJ, Ni ZY, Hu Y, Liang WH, Ou CQ, He JX, Liu L, Shan H, Lei CL, Hui DSC, Du B, Li LJ, Zeng G, Yuen KY, Chen RC, Tang CL, Wang T, Chen PY, Xiang J, Li SY, Wang JL, Liang ZJ, Peng YX, Wei L, Liu Y, Hu YH, Peng P, Wang JM, Liu JY, Chen Z, Li G, Zheng ZJ, Qiu SQ, Luo J, Ye CJ, Zhu SY, Zhong NS 28 2 2020 Clinical characteristics of coronavirus disease 2019 in China. N Engl J Med doi:10.1056/NEJMoa2002032. PMC7092819 32109013

[4] Paules CI, Marston HD, Fauci AS 2020 Coronavirus infections-more than just the common cold. JAMA 323:707. doi:10.1001/jama.2020.0757. 31971553

[5] Lu H, Stratton CW, Tang YW 2020 Outbreak of pneumonia of unknown etiology in Wuhan China: the mystery and the miracle. J Med Virol 92:401–402. doi:10.1002/jmv.25678. 31950516 PMC7166628

[6] Chu DKW, Pan Y, Cheng SMS, Hui KPY, Krishnan P, Liu Y, Ng DYM, Wan CKC, Yang P, Wang Q, Peiris M, Poon L 2020 Molecular diagnosis of a novel coronavirus (2019-nCoV) causing an outbreak of pneumonia. Clin Chem 66:549–555. doi:10.1093/clinchem/hvaa029. 32031583 PMC7108203

[7] Corman VM, Landt O, Kaiser M, Molenkamp R, Meijer A, Chu DKW, Bleicker T, Brunink S, Schneider J, Schmidt ML, Mulders D, Haagmans BL, van der Veer B, van den Brink S, Wijsman L, Goderski G, Romette JL, Ellis J, Zambon M, Peiris M, Goossens H, Reusken C, Koopmans MPG, Drosten C 2020 Detection of 2019 novel coronavirus (2019-nCoV) by real-time RT-PCR. Euro Surveill 25:2000045. doi:10.2807/1560-7917.ES.2020.25.3.2000045. PMC6988269 31992387

[8] Loeffelholz MJ, Tang YW 2020 Laboratory diagnosis of emerging human coronavirus infections — the state of the art. Emerg Microbes Infect 9:747–756. doi:10.1080/22221751.2020.1745095. 32196430 PMC7172701

[9] Chan PK, To WK, Ng KC, Lam RK, Ng TK, Chan RC, Wu A, Yu WC, Lee N, Hui DS, Lai ST, Hon EK, Li CK, Sung JJ, Tam JS 2004 Laboratory diagnosis of SARS. Emerg Infect Dis 10:825–831. doi:10.3201/eid1005.030682. 15200815 PMC3323215

[10] Emery SL, Erdman DD, Bowen MD, Newton BR, Winchell JM, Meyer RF, Tong S, Cook BT, Holloway BP, McCaustland KA, Rota PA, Bankamp B, Lowe LE, Ksiazek TG, Bellini WJ, Anderson LJ 2004 Real-time reverse transcription-polymerase chain reaction assay for SARS-associated coronavirus. Emerg Infect Dis 10:311–316. doi:10.3201/eid1002.030759. 15030703 PMC3322901

[11] Pan Y, Zhang D, Yang P, Poon LLM, Wang Q 2020 Viral load of SARS-CoV-2 in clinical samples. Lancet Infect Dis 24:30113–30114. doi:10.1016/S1473-3099(20)30113-4. PMC7128099 32105638

[12] Zou L, Ruan F, Huang M, Liang L, Huang H, Hong Z, Yu J, Kang M, Song Y, Xia J, Guo Q, Song T, He J, Yen HL, Peiris M, Wu J 2020 SARS-CoV-2 viral load in upper respiratory specimens of infected patients. N Engl J Med 382:1177–1179. doi:10.1056/NEJMc2001737. 32074444 PMC7121626

[13] To KK, Tsang OT, Leung WS, Tam AR, Wu TC, Lung DC, Yip CC, Cai JP, Chan JM, Chik TS, Lau DP, Choi CY, Chen LL, Chan WM, Chan KH, Ip JD, Ng AC, Poon RW, Luo CT, Cheng VC, Chan JF, Hung IF, Chen Z, Chen H, Yuen KY 2020 Temporal profiles of viral load in posterior oropharyngeal saliva samples and serum antibody responses during infection by SARS-CoV-2: an observational cohort study. Lancet Infect Dis 23:30196–30191. 10.1016/S1473-3099(20)30196-1 PMC7158907 32213337

[14] Wolfel R, Corman VM, Guggemos W, Seilmaier M, Zange S, Müller MA, Niemeyer D, Jones TC, Vollmar P, Rothe C, Hoelscher M, Bleicker T, Brunink S, Schneider J, Ehmann R, Zwirglmaier K, Drosten C, Wendtner C 1 4 2020 Virological assessment of hospitalized patients with COVID-2019. Nature doi:10.1038/s41586-020-2196-x. 32235945

[15] Kim C, Ahmed JA, Eidex RB, Nyoka R, Waiboci LW, Erdman D, Tepo A, Mahamud AS, Kabura W, Nguhi M, Muthoka P, Burton W, Breiman RF, Njenga MK, Katz MA 2011 Comparison of nasopharyngeal and oropharyngeal swabs for the diagnosis of eight respiratory viruses by real-time reverse transcription-PCR assays. PLoS One 6:e21610. doi:10.1371/journal.pone.0021610. 21738731 PMC3128075

[16] Wang W, Xu Y, Gao R, Lu R, Han K, Wu G, Tan W 11 3 2020 Detection of SARS-CoV-2 in different types of clinical specimens. JAMA doi:10.1001/jama.2020.3786. PMC7066521 32159775

[17] Druce J, Garcia K, Tran T, Papadakis G, Birch C 2012 Evaluation of swabs, transport media, and specimen transport conditions for optimal detection of viruses by PCR. J Clin Microbiol 50:1064–1065. doi:10.1128/JCM.06551-11. 22205810 PMC3295134

[18] Li Q, Guan X, Wu P, Wang X, Zhou L, Tong Y, Ren R, Leung KSM, Lau EHY, Wong JY, Xing X, Xiang N, Wu Y, Li C, Chen Q, Li D, Liu T, Zhao J, Li M, Tu W, Chen C, Jin L, Yang R, Wang Q, Zhou S, Wang R, Liu H, Luo Y, Liu Y, Shao G, Li H, Tao Z, Yang Y, Deng Z, Liu B, Ma Z, Zhang Y, Shi G, Lam TTY, Wu JTK, Gao GF, Cowling BJ, Yang B, Leung GM, Feng Z 2020 Early transmission dynamics in Wuhan, China, of novel coronavirus-infected pneumonia. N Engl J Med 382:1199–1207. doi:10.1056/NEJMoa2001316. 31995857 PMC7121484

[19] Goff J, Rowe A, Brownstein JS, Chunara R 2015 Surveillance of acute respiratory infections using community-submitted symptoms and specimens for molecular diagnostic testing. PLoS Curr 7:ecurrents.outbreaks.0371243baa7f3810ba1279e30b96d3b6. doi:10.1371/currents.outbreaks.0371243baa7f3810ba1279e30b96d3b6. PMC4455990 26075141

[20] To KK, Lu L, Yip CC, Poon RW, Fung AM, Cheng A, Lui DH, Ho DT, Hung IF, Chan KH, Yuen KY 2017 Additional molecular testing of saliva specimens improves the detection of respiratory viruses. Emerg Microbes Infect 6:e49. doi:10.1038/emi.2017.35. 28588283 PMC5520312

[21] To KK, Tsang OT, Chik-Yan Yip C, Chan KH, Wu TC, Chan JMC, Leung WS, Chik TS, Choi CY, Kandamby DH, Lung DC, Tam AR, Poon RW, Fung AY, Hung IF, Cheng VC, Chan JF, Yuen KY 12 2 2020 Consistent detection of 2019 novel coronavirus in saliva. Clin Infect Dis doi:10.1093/cid/ciaa149. PMC7108139 32047895

[22] Wang WK, Chen SY, Liu IJ, Chen YC, Chen HL, Yang CF, Chen PJ, Yeh SH, Kao CL, Huang LM, Hsueh PR, Wang JT, Sheng WH, Fang CT, Hung CC, Hsieh SM, Su CP, Chiang WC, Yang JY, Lin JH, Hsieh SC, Hu HP, Chiang YP, Wang JT, Yang PC, Chang SC, SARS Research Group of the National Taiwan University/National Taiwan University Hospital. 2004 Detection of SARS-associated coronavirus in throat wash and saliva in early diagnosis. Emerg Infect Dis 10:1213–1219. doi:10.3201/eid1007.031113. 15324540 PMC3323313

[23] Yu F, Yan L, Wang N, Yang S, Wang L, Tang Y, Gao G, Wang S, Ma C, Xie R, Wang F, Tan C, Zhu L, Guo Y, Zhang F 28 3 2020 Quantitative detection and viral load analysis of SARS-CoV-2 in infected patients. Clin Infect Dis doi:10.1093/cid/ciaa345. PMC7184442 32221523

[24] Young BE, Ong SWX, Kalimuddin S, Low JG, Tan SY, Loh J, Ng OT, Marimuthu K, Ang LW, Mak TM, Lau SK, Anderson DE, Chan KS, Tan TY, Ng TY, Cui L, Said Z, Kurupatham L, Chen MI, Chan M, Vasoo S, Wang LF, Tan BH, Lin RTP, Lee VJM, Leo YS, Lye DC, Singapore 2019 Novel Coronavirus Outbreak Research Team. 3 3 2020 Epidemiologic features and clinical course of patients infected with SARS-CoV-2 in Singapore. JAMA doi:10.1001/jama.2020.3204. PMC7054855 32125362

[25] Zhang W, Du RH, Li B, Zheng XS, Yang XL, Hu B, Wang YY, Xiao GF, Yan B, Shi ZL, Zhou P 2020 Molecular and serological investigation of 2019-nCoV infected patients: implication of multiple shedding routes. Emerg Microbes Infect 9:386–389. doi:10.1080/22221751.2020.1729071. 32065057 PMC7048229

[26] Cheng PK, Wong DA, Tong LK, Ip SM, Lo AC, Lau CS, Yeung EY, Lim WW 2004 Viral shedding patterns of coronavirus in patients with probable severe acute respiratory syndrome. Lancet 363:1699–1700. doi:10.1016/S0140-6736(04)16255-7. 15158632 PMC7112423

[27] Isakbaeva ET, Khetsuriani N, Beard RS, Peck A, Erdman D, Monroe SS, Tong S, Ksiazek TG, Lowther S, Pandya-Smith I, Anderson LJ, Lingappa J, Widdowson MA, SARS Investigation Group. 2004 SARS-associated coronavirus transmission, United States. Emerg Infect Dis 10:225–231. doi:10.3201/eid1002.030734. 15030687 PMC3322913

[28] Leung WK, To KF, Chan PK, Chan HL, Wu AK, Lee N, Yuen KY, Sung JJ 2003 Enteric involvement of severe acute respiratory syndrome-associated coronavirus infection. Gastroenterology 125:1011–1017. doi:10.1016/s0016-5085(03)01215-0. 14517783 PMC7126982

[29] Munster VJ, Koopmans M, van Doremalen N, van Riel D, de Wit E 2020 A novel coronavirus emerging in China—key questions for impact assessment. N Engl J Med 382:692–694. doi:10.1056/NEJMp2000929. 31978293

[30] Shi X, Gong E, Gao D, Zhang B, Zheng J, Gao Z, Zhong Y, Zou W, Wu B, Fang W, Liao S, Wang S, Xie Z, Lu M, Hou L, Zhong H, Shao H, Li N, Liu C, Pei F, Yang J, Wang Y, Han Z, Shi X, Zhang Q, You J, Zhu X, Gu J 2005 Severe acute respiratory syndrome associated coronavirus is detected in intestinal tissues of fatal cases. Am J Gastroenterol 100:169–176. doi:10.1111/j.1572-0241.2005.40377.x. 15654797

[31] Xu D, Zhang Z, Jin L, Chu F, Mao Y, Wang H, Liu M, Wang M, Zhang L, Gao GF, Wang FS 2005 Persistent shedding of viable SARS-CoV in urine and stool of SARS patients during the convalescent phase. Eur J Clin Microbiol Infect Dis 24:165–171. doi:10.1007/s10096-005-1299-5. 15789222 PMC7088045

[32] Yeo C, Kaushal S, Yeo D 2020 Enteric involvement of coronaviruses: is faecal-oral transmission of SARS-CoV-2 possible? Lancet Gastroenterol Hepatol 5:335–337. doi:10.1016/S2468-1253(20)30048-0. 32087098 PMC7130008

[33] Blow JA, Dohm DJ, Negley DL, Mores CN 2004 Virus inactivation by nucleic acid extraction reagents. J Virol Methods 119:195–198. doi:10.1016/j.jviromet.2004.03.015. 15158603

[34] Burton JE, Easterbrook L, Pitman J, Anderson D, Roddy S, Bailey D, Vipond R, Bruce CB, Roberts AD 2017 The effect of a non-denaturing detergent and a guanidinium-based inactivation agent on the viability of Ebola virus in mock clinical serum samples. J Virol Methods 250:34–40. doi:10.1016/j.jviromet.2017.09.020. 28941617

[35] Kumar M, Mazur S, Ork BL, Postnikova E, Hensley LE, Jahrling PB, Johnson R, Holbrook MR 2015 Inactivation and safety testing of Middle East respiratory syndrome coronavirus. J Virol Methods 223:13–18. doi:10.1016/j.jviromet.2015.07.002. 26190637 PMC4555185

[36] Duan X, Wang X, Yu P, Liu W, Li X, Zhang L, Zhang G, Tang H, Chen Q, Wu X, Tao Z 2020 Effect of virus inactivation on weak positive results of nucleic acid test for 2019 novel coronavirus. Chin J Lab Med http://rs.yiigle.com/yufabiao/1184369.htm. (In Chinese.)

[37] Nie S, Roth RB, Stiles J, Mikhlina A, Lu X, Tang YW, Babady NE 2014 Evaluation of Alere i Influenza A&B for rapid detection of influenza viruses A and B. J Clin Microbiol 52:3339–3344. doi:10.1128/JCM.01132-14. 24989611 PMC4313160

[38] Wang H, Deng J, Tang YW 2018 Profile of the Alere i Influenza A & B assay: a pioneering molecular point-of-care test. Expert Rev Mol Diagn 18:403–409. doi:10.1080/14737159.2018.1466703. 29688086 PMC6153442

[39] Ling L, Kaplan SE, Lopez JC, Stiles J, Lu X, Tang YW 2017 Parallel validation of three molecular devices for simultaneous detection and identification of influenza A and B and respiratory syncytial viruses. J Clin Microbiol 56:e01691-17. doi:10.1128/JCM.01691-17. PMC5824037 29263204

[40] Chen Y, Chan KH, Hong C, Kang Y, Ge S, Chen H, Wong EY, Joseph S, Patteril NG, Wernery U, Xia N, Lau SK, Woo PC 2016 A highly specific rapid antigen detection assay for on-site diagnosis of MERS. J Infect 73:82–84. doi:10.1016/j.jinf.2016.04.014. 27144915 PMC7127149

[41] Lau SK, Woo PC, Wong BH, Tsoi HW, Woo GK, Poon RW, Chan KH, Wei WI, Peiris JS, Yuen KY 2004 Detection of severe acute respiratory syndrome (SARS) coronavirus nucleocapsid protein in SARS patients by enzyme-linked immunosorbent assay. J Clin Microbiol 42:2884–2889. doi:10.1128/JCM.42.7.2884-2889.2004. 15243033 PMC446266

[42] Sastre P, Dijkman R, Camunas A, Ruiz T, Jebbink MF, van der Hoek L, Vela C, Rueda P 2011 Differentiation between human coronaviruses NL63 and 229E using a novel double-antibody sandwich enzyme-linked immunosorbent assay based on specific monoclonal antibodies. Clin Vaccine Immunol 18:113–118. doi:10.1128/CVI.00355-10. 21084464 PMC3019789

[43] Liu IJ, Chen PJ, Yeh SH, Chiang YP, Huang LM, Chang MF, Chen SY, Yang PC, Chang SC, Wang WK, SARS Research Group of the National Taiwan University College of Medicine-National Taiwan University Hospital. 2005 Immunofluorescence assay for detection of the nucleocapsid antigen of the severe acute respiratory syndrome (SARS)-associated coronavirus in cells derived from throat wash samples of patients with SARS. J Clin Microbiol 43:2444–2448. doi:10.1128/JCM.43.5.2444-2448.2005. 15872279 PMC1153760

[44] Sizun J, Arbour N, Talbot PJ 1998 Comparison of immunofluorescence with monoclonal antibodies and RT-PCR for the detection of human coronaviruses 229E and OC43 in cell culture. J Virol Methods 72:145–152. doi:10.1016/s0166-0934(98)00013-5. 9694322 PMC7119642

[45] Diao B, Wen K, Chen J, Liu Y, Yuan Z, Han C, Chen J, Pan Y, Chen L, Dan Y, Wang J, Chen Y, Deng G, Zhou H, Wu Y 13 3 2020 Diagnosis of acute respiratory syndrome coronavirus 2 infection by detection of nucleocapsid protein. medRxiv https://www.medrxiv.org/content/10.1101/2020.03.07.20032524v1.

[46] Chen X, Zhou B, Li M, Liang X, Wang H, Yang G, Wang H, Le X 2004 Serology of severe acute respiratory syndrome: implications for surveillance and outcome. J Infect Dis 189:1158–1163. doi:10.1086/380397. 15031782 PMC7110198

[47] Chan CM, Tse H, Wong SS, Woo PC, Lau SK, Chen L, Zheng BJ, Huang JD, Yuen KY 2009 Examination of seroprevalence of coronavirus HKU1 infection with S protein-based ELISA and neutralization assay against viral spike pseudotyped virus. J Clin Virol 45:54–60. doi:10.1016/j.jcv.2009.02.011. 19342289 PMC7108224

[48] Briese T, Mishra N, Jain K, Zalmout IS, Jabado OJ, Karesh WB, Daszak P, Mohammed OB, Alagaili AN, Lipkin WI 2014 Middle East respiratory syndrome coronavirus quasispecies that include homologues of human isolates revealed through whole-genome analysis and virus cultured from dromedary camels in Saudi Arabia. mBio 5:e01146-14. doi:10.1128/mBio.01146-14. 24781747 PMC4010836

[49] Chen L, Liu W, Zhang Q, Xu K, Ye G, Wu W, Sun Z, Liu F, Wu K, Zhong B, Mei Y, Zhang W, Chen Y, Li Y, Shi M, Lan K, Liu Y 2020 RNA based mNGS approach identifies a novel human coronavirus from two individual pneumonia cases in 2019 Wuhan outbreak. Emerg Microbes Infect 9:313–319. doi:10.1080/22221751.2020.1725399. 32020836 PMC7033720

[50] Ren LL, Wang YM, Wu ZQ, Xiang ZC, Guo L, Xu T, Jiang YZ, Xiong Y, Li YJ, Li XW, Li H, Fan GH, Gu XY, Xiao Y, Gao H, Xu JY, Yang F, Wang XM, Wu C, Chen L, Liu YW, Liu B, Yang J, Wang XR, Dong J, Li L, Huang CL, Zhao JP, Hu Y, Cheng ZS, Liu LL, Qian ZH, Qin C, Jin Q, Cao B, Wang JW 2020 Identification of a novel coronavirus causing severe pneumonia in human: a descriptive study. Chin Med J 11:1. doi:10.1097/CM9.0000000000000722. PMC7147275 32004165

[51] Wu F, Zhao S, Yu B, Chen YM, Wang W, Song ZG, Hu Y, Tao ZW, Tian JH, Pei YY, Yuan ML, Zhang YL, Dai FH, Liu Y, Wang QM, Zheng JJ, Xu L, Holmes EC, Zhang YZ 2020 A new coronavirus associated with human respiratory disease in China. Nature 579:265–269. doi:10.1038/s41586-020-2008-3. 32015508 PMC7094943

[52] Zhou P, Yang XL, Wang XG, Hu B, Zhang L, Zhang W, Si HR, Zhu Y, Li B, Huang CL, Chen HD, Chen J, Luo Y, Guo H, Jiang RD, Liu MQ, Chen Y, Shen XR, Wang X, Zheng XS, Zhao K, Chen QJ, Deng F, Liu LL, Yan B, Zhan FX, Wang YY, Xiao GF, Shi ZL 2020 A pneumonia outbreak associated with a new coronavirus of probable bat origin. Nature 579:270–273. doi:10.1038/s41586-020-2012-7. 32015507 PMC7095418

[53] Holshue ML, DeBolt C, Lindquist S, Lofy KH, Wiesman J, Bruce H, Spitters C, Ericson K, Wilkerson S, Tural A, Diaz G, Cohn A, Fox L, Patel A, Gerber SI, Kim L, Tong S, Lu X, Lindstrom S, Pallansch MA, Weldon WC, Biggs HM, Uyeki TM, Pillai SK, Washington State 2019-nCoV Case Investigation Team. 2020 First case of 2019 novel coronavirus in the United States. N Engl J Med 382:929–936. doi:10.1056/NEJMoa2001191. 32004427 PMC7092802

[54] Rothe C, Schunk M, Sothmann P, Bretzel G, Froeschl G, Wallrauch C, Zimmer T, Thiel V, Janke C, Guggemos W, Seilmaier M, Drosten C, Vollmar P, Zwirglmaier K, Zange S, Wolfel R, Hoelscher M 2020 Transmission of 2019-nCoV infection from an asymptomatic contact in Germany. N Engl J Med 382:970–971. doi:10.1056/NEJMc2001468. 32003551 PMC7120970

[55] Chan JF, Yuan S, Kok KH, To KK, Chu H, Yang J, Xing F, Liu J, Yip CC, Poon RW, Tsoi HW, Lo SK, Chan KH, Poon VK, Chan WM, Ip JD, Cai JP, Cheng VC, Chen H, Hui CK, Yuen KY 2020 A familial cluster of pneumonia associated with the 2019 novel coronavirus indicating person-to-person transmission: a study of a family cluster. Lancet 395:514–523. doi:10.1016/S0140-6736(20)30154-9. 31986261 PMC7159286

[56] Ai JW, Zhang Y, Zhang HC, Xu T, Zhang WH 2020 Era of molecular diagnosis for pathogen identification of unexplained pneumonia, lessons to be learned. Emerg Microbes Infect 9:597–600. doi:10.1080/22221751.2020.1738905. 32174267 PMC7144283

[57] Cui J, Li F, Shi ZL 2019 Origin and evolution of pathogenic coronaviruses. Nat Rev Microbiol 17:181–192. doi:10.1038/s41579-018-0118-9. 30531947 PMC7097006

[58] Lu R, Zhao X, Li J, Niu P, Yang B, Wu H, Wang W, Song H, Huang B, Zhu N, Bi Y, Ma X, Zhan F, Wang L, Hu T, Zhou H, Hu Z, Zhou W, Zhao L, Chen J, Meng Y, Wang J, Lin Y, Yuan J, Xie Z, Ma J, Liu WJ, Wang D, Xu W, Holmes EC, Gao GF, Wu G, Chen W, Shi W, Tan W 2020 Genomic characterisation and epidemiology of 2019 novel coronavirus: implications for virus origins and receptor binding. Lancet 395:565–574. doi:10.1016/S0140-6736(20)30251-8. 32007145 PMC7159086

[59] Chan JF, Yip CC, To KK, Tang TH, Wong SC, Leung KH, Fung AY, Ng AC, Zou Z, Tsoi HW, Choi GK, Tam AR, Cheng VC, Chan KH, Tsang OT, Yuen KY 4 3 2020 Improved molecular diagnosis of COVID-19 by the novel, highly sensitive and specific COVID-19-RdRp/Hel real-time reverse transcription-polymerase chain reaction assay validated in vitro and with clinical specimens. J Clin Microbiol doi:10.1128/JCM.00310-20. PMC7180250 32132196

[60] China National Health Commission. 2020. New coronavirus pneumonia prevention and control protocol, 7th ed. National Commission of the People’s Republic of China. (In Chinese.) http://www.nhc.gov.cn/yzygj/s7653p/202003/46c9294a7dfe4cef80dc7f5912eb1989/files/ce3e6945832a438eaae415350a8ce964.pdf.

[61] Kam KQ, Yung CF, Cui L, Lin Tzer Pin R, Mak TM, Maiwald M, Li J, Chong CY, Nadua K, Tan NWH, Thoon KC 28 2 2020 A well infant with coronavirus disease 2019 (COVID-19) with high viral load. Clin Infect Dis doi:10.1093/cid/ciaa201. PMC7358675 32112082

[62] Liu Y, Yan LM, Wan L, Xiang TX, Le A, Liu JM, Peiris M, Poon LLM, Zhang W 19 3 2020 Viral dynamics in mild and severe cases of COVID-19. Lancet Infect Dis doi:10.1016/S1473-3099(20)30232-2. PMC7158902 32199493

[63] Cheng VCC, Wong SC, Chen JHK, Yip CCY, Chuang VWM, Tsang OTY, Sridhar S, Chan JFW, Ho PL, Yuen KY 3 3 2020 Escalating infection control response to the rapidly evolving epidemiology of the coronavirus disease 2019 (COVID-19) due to SARS-CoV-2 in Hong Kong. Infect Control Hosp Epidemiol doi:10.1017/ice.2020.58. PMC7137535 32131908

[64] Lan L, Xu D, Ye G, Xia C, Wang S, Li Y, Xu H 27 2 2020 Positive RT-PCR test results in patients recovered from COVID-19. JAMA doi:10.1001/jama.2020.2783. PMC7047852 32105304

[65] Liu Z, Xiao X, Wei X, Li J, Yang J, Tan H, Zhu J, Zhang Q, Wu J, Liu L 2020 Composition and divergence of coronavirus spike proteins and host ACE2 receptors predict potential intermediate hosts of SARS-CoV-2. J Med Virol 26:25726. doi:10.1002/jmv.25726. PMC7228221 32100877

[66] Yan R, Zhang Y, Li Y, Xia L, Guo Y, Zhou Q 2020 Structural basis for the recognition of the SARS-CoV-2 by full-length human ACE2. Science 367:1444–1448. doi:10.1126/science.abb2762. 32132184 PMC7164635

[67] Chan-Yeung M, Xu RH 2003 SARS: epidemiology. Respirology 8:S9–S14. doi:10.1046/j.1440-1843.2003.00518.x. 15018127 PMC7169193

[68] Liu Y, Eggo RM, Kucharski AJ 2020 Secondary attack rate and superspreading events for SARS-CoV-2. Lancet 395:e47. doi:10.1016/S0140-6736(20)30462-1. 32113505 PMC7158947

[69] Guo L, Ren L, Yang S, Xiao M, Chang D, Yang F, Dela Cruz CS, Wang Y, Wu C, Xiao Y, Zhang L, Han L, Dang S, Xu Y, Yang Q, Xu S, Zhu H, Xu Y, Jin Q, Sharma L, Wang L, Wang J 21 3 2020 Profiling early humoral response to diagnose novel coronavirus disease (COVID-19). Clin Infect Dis doi:10.1093/cid/ciaa310. PMC7184472 32198501

[70] Li Z, Yi Y, Luo X, Xiong N, Liu Y, Li S, Sun R, Wang Y, Hu B, Chen W, Zhang Y, Wang J, Huang B, Lin Y, Yang J, Cai W, Wang X, Cheng J, Chen Z, Sun K, Pan W, Zhan Z, Chen L, Ye F 2020 Development and clinical application of a rapid IgM-IgG combined antibody test for SARS-CoV-2 infection diagnosis. J Med Virol 27:25727. doi:10.1002/jmv.25727. PMC7228300 32104917

